# “Fast track” rehabilitation after gastric cancer resection: experience with 80 consecutive cases

**DOI:** 10.1186/1471-230X-14-147

**Published:** 2014-08-18

**Authors:** Jing-xiang Song, Xiao-huang Tu, Bing Wang, Chen Lin, Zai-zhong Zhang, Li-ying Lin, Lie Wang

**Affiliations:** 1 Department of General Surgery, Fuzhou General Hospital of Nanjing Military Command, No. 156 North Xi’erhuan Road, Fuzhou 350025, Fujian, China

**Keywords:** Gastric cancer, Fast-track surgery, Perioperative treatment, Hospital stay, Morbidity

## Abstract

**Background:**

To evaluate the safety, efficacy and outcomes of fast-track rehabilitation applied to gastric cancer proximal, distal and total gastrectomy.

**Methods:**

Eighty consecutive patients undergoing gastric cancer resection performed by a single surgeon, received perioperative multimodal rehabilitation. Demographic and operative data, gastrointestinal function, postoperative hospital stays, surgical and general complications and mortality were assessed prospectively.

**Results:**

Of the 80 patients (mean age 56.3 years), 10 (12.5%) received proximal subtotal gastrectomy (Billroth I), 38 (47.5%) received distal (Billroth II), and 32 (40%) received total gastrectomy (Roux-en-Y). Mean operative time was 104.9 minutes and intraoperative blood loss was 281.9 ml. Time to first flatus was 2.8 ± 0.5 postoperative days. Patients were discharged at a mean of 5.3 ± 2.2 postoperative days; 30-day readmission rate was 3.8%. In-hospital mortality was 0%; general and surgical complications were both 5%.

**Conclusions:**

Fast-track multimodal rehabilitation is feasible and safe in patients undergoing gastric cancer resection and may reduce time to first flatus and postoperative hospital stays.

## Background

Gastric cancer, the second most common cause of cancer deaths worldwide, accounts for over 8.8% of all cancer-related deaths [1]. The incidence of gastric cancer in Asia is high; it is the most prevalent cancer among males in China and Japan and half of the world’s total number of cases are found in Eastern and South East Asian countries [1]. Although the accepted standard treatment for gastric cancer has been gastrectomy with D2 lymph node dissection, this treatment still has significant morbidity (about 20%) and mortality (3.1%) [2-4]. The comparative advantages in outcomes, perioperative morbidity and long-term survival between total gastrectomy and distal gastrectomy remain controversial [5]. Extended lymphadenectomy (D3 vs. D1] is associated with more complications and higher morbidity than limited lymphadenectomy, but it does not markedly increase mortality [6]. In fact, D2 dissection has been shown to improve survival [7-9], with routine preservation of spleen and pancreas [8].

Total gastrectomy performed for curative or palliative intent has been found to be a safe procedure with acceptable mortality rates (20% across early to late stages of disease) [10], and minimally-invasive laparoscopic gastrectomy was shown to result in more rapid recovery, fewer complications and shortened hospital stays [11,12]. However, the rates of postoperative morbidity after gastric cancer resection remain between 10% and 40%, and postoperative complications such as anastomotic leakage, pleuropulmonary disease, pancreatitis, digestive fistulas, internal bleeding, and bowel obstruction can result in prolonged hospital stays ranging from 8–20 days at high-volume centers [6,7,9,13,14].

Within the last twenty years, the use of safe short-acting anesthesia, pain control, reduction of perioperative stress and the use of minimally invasive laparoscopic surgery have helped to improve postoperative outcomes, primarily based upon a better understanding of perioperative pathophysiology [15-17]. The effort to combine these advantages with revisions of other evidence-based perioperative care principles has been designated “fast-track surgery” [15]. Fast-track surgery (FTS) is an integrated application of laparoscopic vs. open surgery, minimally invasive anesthesia and pain relief, and cooperation between surgeons, surgical nurses and physiotherapists aimed at reducing risk and pain to enhance recovery after surgery [16]. FTS has been applied to various operations, especially colorectal surgeries [17-23], and has significantly reduced postoperative hospital stays in high-risk patients undergoing colonic resection [17] by reducing the commonly known stress responses associated with surgery. Studies examining the outcomes of fast-track colorectal resection showed that postoperative stays were reduced by 2–4 days, with lower complication rates and reduction of total related hospital costs [20-23]. FTS programs in elective open repair of abdominal aneurysm also reduced the incidence of systemic inflammatory response after high risk surgery [24].

To date, most gastrectomy studies have focused on distal subtotal gastrectomy. FTS was recently shown to shorten the duration of flatus, reduce complications and shorten the duration of hospital stay in patients undergoing radical total gastrectomy compared to patients receiving conventional treatment [4]. In this prospective study, we explored the safety and efficacy of FTS in 80 consecutive gastric cancer patients undergoing proximal, distal and total gastrectomy in our institution. We adopted perioperative care regimens from previous reports [15,16], and aimed to achieve three major goals: optimal anesthesia and analgesia, early normal gastrointestinal function based on stepwise food intake and earlier passing of flatus, and early complete mobilization.

## Methods

### Study design and sample

This prospective study analyzed the data of consecutive patients undergoing elective resection of gastric cancer between January 2011 and February 2012 in our institution. Our study population was comprised of Han Chinese from Fuzhou, Putian, Quanzhou and other coastal areas of the Fujian province. Ethnic diversity was therefore not a problem. All patients underwent open surgeries performed by a single surgeon (T.X.H). Patients with emergency surgery, history of chemoradiation within the 6 months preceding surgery, preoperative evidence of distant metastases, additional resection of adjacent organs, ASA score > III or inability to communicate or to understand the purpose of the study were excluded. All data were assessed prospectively. The study protocol was approved by the Ethics Committee of the Fuzhou General Hospital of Nanjing Military Region. Of 92 patients screened initially, 80 patients were finally included after 12 were excluded for: multiple organ resection (4 patients), distal metastases (4 patients), ASA score > III (2 patients), and unable to communicate or understand study purpose (2 patients). All surgical patients provided signed informed consent for their data to be entered into the study without revealing their identities.

### Surgical procedures

All gastric resections were performed in accordance with standardized procedures. The extent of gastric resection was determined as proximal subtotal gastrectomy for cancers of the superior third, distal subtotal gastrectomy for cancers of the lower third and total gastrectomy for cancers of the middle third. A radical lymphadenectomy without splenectomy and pancreatectomy (standard D2 procedure) was performed in all patients undergoing gastrectomy for cancer. Digestive continuity was restored by a Billroth I gastroduodenostomy or Billroth II gastrojejunostomy after partial gastrectomy, and a Roux-en-Y jejunal loop after total gastrectomy (esophagojejunostomy). Discharge criteria consisted of: (1) adequate pain control with oral medication; (2) absence of nausea and or/vomiting; (3) passage of first flatus; (4) ability to tolerate non-elemental diet and soft food; (5) mobilization and self-support, and (6) acceptance of discharge by the patient. Within 24 to 48 hours after discharge, FT patients were contacted by telephone by a specially trained resident (L.C.) to check for complications, and then once weekly until one month after the surgery. All patients were seen at the outpatient department for postoperative examination at a minimum of 10 days postoperatively.

### Protocols for perioperative fast-track rehabilitation

The multimodal protocols for elective fast-track gastric cancer resection are shown in Table 1, including preoperative, intraoperative and postoperative phases, as previously described [15,16]. The protocols include no bowel preparation, no routine use of nasogastric tubes and abdominal drains, and patients received single-shot antibiotic prophylaxis (2.0 g cefoperazone) at the induction of anesthesia. Placement of an epidural catheter at the level of Th8–Th10 was recommended for all patients. An epidural infusion of bupivacaine 0.25% with 3 μg/ml fentanyl at a rate of 5–15 ml/h was started at the end of surgery. Continuous post-operative epidural analgesia was administered via a pump up to 3 postoperative days; the epidural infusion was decreased on postoperative day 2 and discontinued on day 3. All patients also received 500 mg tramadol twice a day orally before discharge.

**Table 1:** Multimodal protocols for elective fast-track gastric cancer surgery**

	
Preoperative phase	
	Scheduling of operation
	Information about FT and informed consent
	Pre-assessment for risk adjustment
	Last meal 6 h before operation
	Last clear drink (10% Glucose 500 ml) 2 h before operation
Intraoperative phase	
	Prophylactic antibiotic (cefoperazone 2 g)
	Placement of thoracic epidural catheter (T8-T10) followed by continuous EDA until POD3
	Combined with general anesthesia
	Restricted intraoperative fluid therapy to 500 cc colloid and 1,500 cc crystalloid infusion
	Use of vasopressor drugs as 1st choice for management of mean blood pressure drop >20% of baseline
	Prophylactic use of odansetron to prevent PONV
	Forced body heating
	No standard use of abdominal drains and nasogastric tube
	Intradermic suture with absorbable suture
Postoperative phase	
	Admit to regular nursing floor via ICU (POD 1)
	Continuous EDA (3d) with tramadol 500 mg po 12 h
	Oral intake of clear liquids (100-150 ml Glucose) 2 h after extubation, followed by stepwise plan from warm clear water to carbohydrate drink to TPF, then to semi-fluids to normal food.
	Adhere to a regimen of frequent small meals.
	Restricted IV fluid administration until complete oral intake
	Enforced mobilization from the day of surgery following a well-defined nursing care program
	Removal of bladder catheter in POD1 morning
	Outpatient clinic; discuss result of histological examination, plan adjuvant therapy if needed (POD 10)

FT = Fluid therapy.

POD = postoperative day.

EDA = epidural analgesia.

ICU = intensive care unit.

PONV = Postoperative nausea and vomiting.

TPF = Commercial brand of an enteral nutritional suspension.

**Adapted from Kehlet et al. [15] and Kehlet et al. [16].

### Postoperative outcomes analysis

Postoperative hospital stays and readmission rates, time to first flatus and use of intravenous fluid during the postoperative procedure were monitored for 30 days after surgery. Complications requiring treatment were recorded during the first 30 postoperative days. General complications were defined as: cardiovascular, pulmonary, urinary tract and other complications. Surgical complications were defined as: wound complications, anastomotic leaks, bowel obstruction and other complications. Perioperative mortality included deaths within the first 30 days after surgery or during the original hospital stay if longer than 30 days. Fistula was defined as a proven leak at water soluble contrast radiographic examination, or a leak of clinical significance necessitating reoperation.

### Statistical analysis

Continuous variables are presented as means and standard deviations. Categorical variables are presented as counts and percentages. All statistical analyses were done using SPSS 17.0 statistics software (SPSS Inc, Chicago, IL).

## Results

### Distribution of patients’ demographic and clinical characteristics

The distribution of patient characteristics is shown in Table 2. The 80 patients included 25 females and 55 males, with a mean age of 56.3 years (56.3 ± 10.6 years) and mean BMI of 22.8 kg/m^2^. There were 24 patients (30%) in ASA class I, 50 patients (62.5%) in ASA class II, and 6 patients (7.5%) in ASA class III. Evaluation of concomitant diseases revealed 13 (16.3%) patients with cardiovascular disease; 10 (12.5%) patients with chronic pulmonary disease; 5 (6.3%) patients with neurologic disease; 6 (7.5%) patients with endocrine disease; and 3 (3.8%) patients with other diseases. The remaining 52 (65%) patients had no concomitant diseases. There were 10 (12.5%) patients who received proximal subtotal gastrectomy (Billroth I); 38 (47.5%) patients who received distal subtotal gastrectomy (Billroth II); and 32 (40%) patients who received total gastrectomy (Roux-en-Y). The tumors in patients receiving Billroth I surgery were located in the middle region of the gastric body. There were no significant differences in demographics or clinical characteristics between the patients who received Billroth I or Billroth II procedures (data not shown). Evaluation of tumor stages revealed 8 (10%) patients at stage I, 46 (57.5%) patients at stage II, and 26(32.5%) patients at stage III. The means of operative time and intraoperative blood loss in the 80 patients were 104.9 minutes and 281.9 ml, respectively (Table 2). We analyzed the basic demographics and clinical characteristics of the patients (47.5%) who received distal subtotal gastrectomy and found no significant differences between this subgroup and the total study population (data not shown).

**Table 2:** Distribution of demographic and clinical characteristics

	**Total (N = 80)**
Age (years)	56.3 ± 10.6
Male	55(68.8%)
BMI (kg/m^2^)	22.8 ± 3.2
ASA Score	
I	24(30.0%)
II	50(62.5%)
III	6(7.5%)
Concomitant diseases	
Cardiovascular disease	13(16.3%)
Chronic pulmonary disease	10(12.5%)
Neurologic disease	5(6.3%)
Endocrine	6(7.5%)
Other diseases	3(3.8%)
Patients without concomitant diseases	52(65.0%)
Type of surgery (reconstruction)	
Proximal subtotal gastrectomy (Billroth I)	10 (12.5%)
Distal subtotal gastrectomy (Billroth II)	38 (47.5%)
Total gastrectomy (Roux-en-Y)	32 (40.0%)
Tumor stage	
I	8 (10.0%)
II	46 (57.5%)
III	26 (32.5%)
Operative time (min)	105.0 ± 13.0
Intraoperative blood loss (ml)	281.9 ± 87.7

ASA: American Society of Anesthesiologists.

BMI = Body mass index.

### Postoperative course and gastrointestinal function

On average, patients were discharged after 5.3 ± 2.2 postoperative days. The mean time for using intravenous fluid was 3.6 ± 0.9 postoperative days. The 30-day inpatient readmission rate was 3.8%. Three patients were readmitted due to wound dehiscence, wound seroma and bowel obstruction. The mean time to first flatus was 2.8 ± 0.5 postoperative days, and time to complete oral intake was 4.3 ± 2.4 postoperative days. There was no significant difference between the Billroth I and Billroth II groups or between patients who received partial or total gastrectomies in the mean time to first flatus or complete oral intake (data not shown). Postoperative nausea and vomiting (PONV) occurred in 2(2.5%) patients and 1(1.3%) patient required nasogastric tube insertion (Table 3).

**Table 3:** Postoperative course and gastrointestinal function

	**Total (N = 80)**
Discharge (POD)	5.3 ± 2.2
Intravenous fluids (POD)	3.6 ± 0.9
Readmission rate (30 days)	3 (3.8%)
Wound dehiscence	1 (1.3%)
Wound seroma	1 (1.3%)
Bowel obstruction	1 (1.3%)
Time to first flatus (POD)	2.8 ± 0.5
Complete oral intake (POD)	4.3 ± 2.4
PONV	2(2.5%)
Insertion of nasogastric tube	1(1.3%)

POD: Postoperative days.

PONV: Postoperative nausea and vomiting.

### Postoperative complications and mortality

Surgical complications were diagnosed in 4 (5%) patients, including 1 with anastomotic leakage, 1 with bowel obstruction, 1 with wound seroma and 1 with wound dehiscence. General complications were diagnosed in 4 (5%) patients, including 1 with myocardial dysfunction, 1 with hypertension, 1 with pneumonia and 1 with urinary retention. Reoperation was necessary in 2 (2.5%) patients, 1 with anastomotic leakage and 1 with wound dehiscence. No deaths occurred within the first 30 days after surgery (Table 4). Serious surgical complications such as anastomotic leakage, or obstruction affected the patient’s recovery of gastrointestinal function and prolonged recovery time and length of hospital stay. However, non-severe complications such as hematoma, or wound dehiscence had little effect on patient rehabilitation. General complications such as high blood pressure, pneumonia, and heart failure did not significantly influence recovery as long as there was timely detection and treatment (data not shown).

**Table 4:** Complications and mortality within the first postoperative month after gastrectomy

	**Total (N = 80)**
Overall complications	8(10.0%)
Surgical complications	4(5.0%)
Anastomotic leakage	1(1.3%)
Bowel obstruction	1(1.3%)
Wound seroma	1(1.3%)
Wound dehiscence	1(1.3%)
General complications	4(5.0%)
Myocardial dysfunction	1(1.3%)
Hypertension	1(1.3%)
Pneumonia	1(1.3%)
Urinary retention	1(1.3%)
Reoperation	2(2.5%)
Anastomotic leakage	1(1.3%)
Wound dehiscence	1(1.3%)
Perioperative mortality	0(0.0%)

### Data comparison between the present study and other recent published studies

Since we were unable to enroll a cohort of control patients due to unavailability of complete medical records for this population, we compared our data with results from five other recently published studies [24-28]. Our comparison showed that the mean operation time in this study was the shortest (104.9 vs.159.9, 213.9, 213.0, 199.8, 226.4 minutes); the mean blood loss was the highest (281.9 vs. 230.1, 201.7, 257.8, 200.4 mL); the mean hospital stay was the shortest (5.28 vs. 7.0, 17.4, 11.1, 17.2 days); the mean time to first flatus was the shortest (2.83 vs. 3.1, 3.2, 4.4, 4.0 days); and the mean time to full oral intake was the shortest (4.31 vs. 5.1, 5.6, 5.5 days) (Table 5).

**Table 5:** Comparison of statistical data from published studies and the present study

**Authors**	**OG cases (n)**	**Operation time (mins)**	**Blood loss (mL)**	**Hospital stay (days)**	**Time to first flatus**	**Time to normal diet (days)**
Feng et al. [4]	122					
	61 FTS	226.1 ± 65.9	230.5 ± 171.8	5.7 ± 1.2	60.9 ± 24.4 h	
	61 Con	242.4 ± 72.9	221.2 ± 122.5	7.1 ± 2.1	79.0 ± 20.3 h	
Chen et al. [25]	112	213.0 ± 54.7	201.7 ± 235.3	17.4 ± 5.0	3.2 ± 1.1 days	Fluid diet: 5.1 ± 1.8
						Soft diet: 10.3 ± 1.6
Chun et al. [26]	67	159.9 ± 39.0	-	7.0 ± 1.6	3.1 ± 0.8 days	-
Cui et al. [27]	78	213.9 ± 37.6	230.1 ± 96.8	-	-	-
Lin et al. [28]	83	226.4 ± 63.5	200.4 ± 218.3	17.2 ± 5.0	4.0 ± 1.0 days	5.5 ± 2.3
Wang et al. [12]	54	199.8 ± 40.8	257.8 ± 151.0	11.1 ± 4.1	4.4 ± 1.5 days	Fluid intake: 5.6 ± 2.1
						Semifluid intake: 7.4 ± 2.4
The present study	80	104.9 ± 13.0	281.9 ± 87.7	5.3 ± 2.2	2.8 ± 0.5 days	4.31 ± 2.43

OG: Open gastrectomy; FTS: Fast Track Surgery; Con: Conventional surgery.

## Discussion

In this study, we demonstrated the safety and feasibility of FTS in patients undergoing proximal, distal and total gastrectomy. Patients’ gastrointestinal function was restored rapidly and postoperative hospital stays were reduced to a mean of 5.3 days, compared to other studies with more conventional perioperative care [12,25-28]. The mean operative time was 104.9 minutes and time to first flatus was 2.8 postoperative days. Patients were discharged at a mean of 5.2 postoperative days and the 30-day readmission rate was 3.75%. The rates of general as well as surgical complications were both 5%. The morbidity (10%) in this study compared favorably with other studies that utilized conventional perioperative care [6-8,10]. Notably, in-hospital mortality was 0%; no deaths occurred within the first 30 days after surgery.

FTS is the implementation of a combination of preoperative, intraoperative and postoperative measures to achieve optimal outcomes in surgical procedures. FTS is especially useful for procedures such as gastrectomy in regions like Fujian, China, which has a high incidence of gastric cancer. Measures such as improved operative skills, and shortened operative times would reduce surgical stress and promote rapid recovery in patients undergoing surgical procedures. The traditional radical gastrectomy perioperative procedure includes 1) fasting 12 hours prior to surgery, 2) stopping fluid intake 6 hours prior to surgery, 3) bowel preparation (enemas and oral antibiotics), 4) administration of general anesthesia, 5) nasogastric tube and peritoneal drainage tube placement 6) administration of conventional intravenous analgesics, 7) resumption of diet after the first flatus, and 8) resumption of ambulation 2–3 days after surgery. Several factors are responsible for increasing recovery time and hospital stays associated with gastric surgery. Postoperative ileus can interfere with resumption of gastrointestinal function and time to restoration of full activities, which may both delay discharge [29]. Interruption of bowel peristalsis results mainly from the direct effect of surgical stress on sympathetic tone and activation of inhibitory reflexes. Several studies have demonstrated that administration of local anesthetics into the thoracolumbar epidural area can decrease the sympathetic tone, allowing the parasympathetic tone to increase and thereby promoting peristalsis [30-32]. The presence of postoperative pain also is one of the most important factors that delays postoperative recovery, and provision of optimal analgesia with no motor blockade facilitates oral feeding and minimizes immobility [33,34]. FTS aims to improve outcomes and promote early discharge by emphasizing preoperative patient education, shortening the duration of preoperative fasting, supplying preoperative carbohydrates, controlling pain sufficiently without opioids, providing early ambulation, and quickly advancing the return to a normal diet [15,16].

Several studies showed that fast-track programs resulted in significantly reduced postoperative hospital stays for colonic and gastric surgeries [17,23,35-38]. Although incomplete implementation was one of the difficulties of FTS, re-operation rates were comparable with conventional surgery [39]. FTS principles applied to D2 gastrectomy were shown to be safe and efficient and could speed the recovery of gut function and shorten postoperative hospital stays [40]. Similarly, implementation of fast-track principles for gastric surgery resulted in a reduced stress response, shorter hospital stays and faster recovery [40-43].

Since we did not have a direct comparison with a control group of patients who underwent conventional treatment, we compared our data with the control groups from previous reports. In this study, FTS patients had a mean hospital stay of 5.28 days, which was significantly less than studies where patients underwent gastric cancer resection with conventional care [8]. Hospital stays in other studies where the patients received conventional treatment have been reported as 17.4 ± 5.0 days [25], 7.0 ± 1.6 days [26], 17.2 ± 5.0 days [28], and 11.1 ± 4.1 days [12]. Our data were consistent with a recent study showing that hospital stays were shortened from 7.1 ± 2.1 days in the conventional group to 5.7 ± 1.2 days in the FTS group after radical total gastrectomy [4].

In the present study, time to first flatus was 2.8 days. This was lower compared to the control groups in the studies that we used for comparative purposes, where the times to first flatus were 3.1 ± 0.8 days [26], 4.0 ± 1 days [28], 3.2 ± 1.1 days [25], 4.4 ± 1.5 days [12]. Other studies showed that time to first flatus ranged from 3.7- 4.5 days [44,45]. However, our data were in agreement with a similar study where patients who underwent radical total gastrectomy in the FTS group had a significantly shorter time to first flatus compared to the conventional treatment group (60.9 + 24.4 hours vs. 79.0 + 20.0 hours) [4].

In this study, patients were able to resume complete oral intake on a median of 4 days postoperatively. This was faster compared to other studies where patients who received conventional treatment resumed fluid diets at 5.1 ± 1.8 days [25], 5.5 ± 2.3 days [28], or 5.6 ± 2.1 days [12]. We suggest that continuous epidural anesthesia and efficient pain control in the FTS regimen may be key elements in the fast recovery of gastrointestinal function and early return to a normal diet, thereby preventing postoperative ileus [19]. There was no significant difference between the type of gastrectomy performed (partial gastrectomy vs. total gastrectomy) and the time to first flatus or complete oral intake. However, patients with partial resection were more careful with food intake, possibly due to fears of the effects of an empty stomach.

Early and complete mobilization of patients is achieved in FTS rehabilitation by quick removal of the urinary catheter, no routine use of nasogastric tubes and abdominal drains, and optimized postoperative pain management [15,16]. In one study, patients were out of bed for a median of 10 hours on the first day after surgery, increasing to 14 hours from day 2 [19], contributing to the overall earlier recovery of gastrointestinal function. After gastrectomy, nasogastric decompression and abdominal drains were traditionally considered necessary to prevent the consequences of postoperative ileus and anastomotic leakage or leaking from the duodenal stump. However, several recent prospective studies have suggested that the use of a nasogastric tube had no significant effect on morbidity or mortality but significantly prolonged the median postoperative hospital stay after gastrectomy for gastric cancer [44-47]. Recent prospective trials also demonstrated that routinely placed drains did not reduce mortality or morbidity [48-50], and that fistulas can be treated with surgical irrigation and drainage. In the present study, only one patient required reoperation for an esophagojejunostomy leak, and one patient required the insertion of a nasogastric tube. We suggest that the practice of not using nasogastic tubes and abdominal drains routinely is both practical and justified and is a crucial factor for the success of the fast-track concept applied to gastric surgery.

The overall incidence of complications and mortality in this study (10% and 0%, respectively) were consistent with other reports [13,14]. Furthermore, the incidence of readmission among our FTS patients was significantly lower (3.8% vs. 16%) compared to those receiving conventional care for high-risk surgery [51]. These observations suggest physiological mechanisms that may be responsible in part for the reduced incidence of postoperative complications and other benefits of fast-track surgery. Interestingly, we showed that in patients who received Billroth I surgery, long-term side effects and the incidence of gastrointestinal complications were lower than in patients who received Billroth II or the esophagojejunostomy procedures (data not shown).

It is important to note that postoperative outcome is affected by a number of factors including surgical technique and postoperative care. In order to control for these factors, all the surgical procedures described in the present study were performed by a single surgeon (T.X.H), who has worked in the field of gastric surgery for more than a decade. There was no difference in surgical procedure compared with previously used techniques. Additionally, the nursing team was stable, all perioperative care procedures were in compliance with standard regulations, and there has been no significant change in the quality of care over the past decade.

Our study has several limitations, including the relatively small sample, lack of randomization, short follow-up time, and the fact that some factors were only descriptive and not quantitative. It will be an important goal of future studies to perform data mining in order to investigate the association between specific pre/intra/postoperative variables and the outcome of the procedure. In this study, the operation time was significantly lower compared to other studies. It will be interesting to evaluate if variables such as operation time, blood loss, and early postoperative resumption of mobilization could be independent factors affecting the outcome of FTS. The major limitation of the study was the unavailability of complete medical records for a control group of patients who received conventional treatment over a similar time frame. We recognize the importance of comparing data from the FTS approach with data from patients undergoing conventional treatment in the same hospital, at the same time period, or comparing our data with historical controls. In order to overcome this limitation, we compared the main results of the study with results from five recent studies that applied conventional perioperative care. Large, multicenter, randomized controlled clinical trials are needed to evaluate the fast-track approach further in gastric cancer patients. It will also be interesting to explore whether laparoscopic fast-track gastric resection may demonstrate significant additional improvements in outcomes.

## Conclusions

A fast-track perioperative care program is feasible and safe in patients undergoing gastric cancer resection and reduces time to first flatus and time to normal diet while shortening post-operative recovery time and hospital stay.

## Competing interests

The authors declare that they have no competing interests.

## Authors’ contributions

JXS: study design, literature research, clinical studies, manuscript preparation, and manuscript editing. XHT: study concepts, definition of intellectual content, clinical studies, and manuscript review. BW: clinical studies, and data acquisition. CL: clinical studies, statistical analysis, and data acquisition. ZZZ: clinical studies, data analysis, and manuscript preparation. LYL: clinical studies, and data acquisition. LW: guarantor of integrity of the entire study, study concepts, study design, definition of intellectual content, and manuscript review. All authors read and approved the final manuscript.

## Pre-publication history

The pre-publication history for this paper can be accessed here:

http://www.biomedcentral.com/1471-230X/14/147/prepub
